# Effect of *VaMyb40* and *VaMyb60* Overexpression on Stilbene Biosynthesis in Cell Cultures of Grapevine *Vitis amurensis* Rupr.

**DOI:** 10.3390/plants11151916

**Published:** 2022-07-24

**Authors:** Alexey A. Ananev, Andrey R. Suprun, Olga A. Aleynova, Nikolay N. Nityagovsky, Zlata V. Ogneva, Alexandra S. Dubrovina, Konstantin V. Kiselev

**Affiliations:** Laboratory of Biotechnology, Federal Scientific Center of the East Asia Terrestrial Biodiversity, FEB RAS, 690022 Vladivostok, Russia; lexx-dance@mail.ru (A.A.A.); suprun.hi@gmail.com (A.R.S.); aleynova@biosoil.ru (O.A.A.); niknit1996@gmail.com (N.N.N.); zlata.v.ogneva@gmail.com (Z.V.O.); dubrovina@biosoil.ru (A.S.D.)

**Keywords:** transcription factors, MYB R2R3, resveratrol, anthocyanins, plant cell cultures, transgenic cells

## Abstract

Stilbenes are plant defense compounds known to rapidly accumulate in grapevine and some other plant species in response to microbial infection and several abiotic stresses. Stilbenes have attracted considerable attention due to valuable biological effects with multi-spectrum therapeutic application. However, there is a lack of information on natural signaling pathways and transcription factors regulating stilbene biosynthesis. It has been previously shown that MYB R2R3 transcription factor genes *VaMyb40* and *VaMyb60* were up-regulated in cell cultures of wild-growing grapevine *Vitis amurensis* Rupr. in response to UV irradiation. In this study, the effects of *VaMyb40* or *VaMyb60* overexpression in cell cultures of *V. amurensis* on their capability to produce stilbenes were investigated. Overexpression of the *VaMyb60* gene led to a considerable increase in the content of stilbenes in three independently transformed transgenic lines in 5.9–13.9 times, while overexpression of the *VaMyb40* gene also increased the content of stilbenes, although to a lesser extent (in 3.4–4.0 times) in comparison with stilbene levels in the empty vector-transformed calli. Stilbene content and stilbene production in the *VaMyb60*-transgenic calli reached 18.8 mg/g of dry weight (DW) and 150.8 mg/L, respectively. Using HPLC analysis, we detected eight individual stilbenes: *t*-resveratrol diglucoside, *t*-piceid, *t*-resveratrol, ε-viniferin, δ-viniferin, *cis*-resveratrol, *cis*-piceid, *t*-piceatannol. *T*-resveratrol prevailed over other stilbenoid compounds (53.1–89.5% of all stilbenes) in the *VaMyb*-overexpressing cell cultures. Moreover, the *VaMyb40-* and *VaMyb60*-transformed calli were capable of producing anthocyanins up to 0.035 mg/g DW, while the control calli did not produce anthocyanins. These findings show that the *VaMyb40* and *VaMyb60* genes positively regulate the stilbene biosynthesis as strong positive transcription regulators and can be used in biotechnological applications for stilbene production or high-quality viticulture and winemaking.

## 1. Introduction

Stilbenes are plant natural phytoalexins with antifungal and insecticide activities whose synthesis is induced upon pathogen attack and other environmental stresses in grapevine and other plant species from a number of unrelated plant families [1,2,3]. Plant stilbenes have become a focus of multiple studies in medicine as promising agents with diverse biological activities, such as antitumor, cardioprotective, antiangiogenic and immunomodulatory properties [4,5,6,7]. *Trans*-resveratrol or *t*-resveratrol (3,5,4′-trihydroxystilbene) is the most prominent and well-studied stilbene with a great variety of pharmacological effects that are perspective for applications in pharmaceutical and cosmetic industries [8,9].

The stilbene biosynthetic pathway diverges from the phenylpropanoid/polymalonate pathway, and the last step of this pathway is being catalyzed by stilbene synthases [3,10]. Stilbene synthases (STS; EC 2.3.1.95) are members of the type III polyketide synthases family and catalyze synthesis of the parent monomeric stilbenes, such as resveratrol or pinosylvin, from coenzyme A-esters of cinnamic acid derivatives and three malonyl-CoA units in a single reaction [10]. The monomeric stilbenes may undergo oxidative dimerization to form stilbene oligomers (viniferins, ampelopsin, or hopeaphenol), glycosylation to form glycosylated stilbenes (piceid or astringin), methoxylation to form *O*-methylated stilbenes (pterostilbene or pinosylvin-3-O-methyl ether), or isoprenylation to form isoprenylated stilbenes (arachidin-3) [11,12,13]. A number of studies show that stilbene production is relatively low under natural conditions in most plant species and strongly depend on stage of development and environmental conditions [3]. Various strategies have been developed to enhance stilbene production levels and to control the composition of produced stilbenes, e.g., plant cell culture elicitation or genetic engineering approaches [3]. Studying the molecular and genetic mechanisms of stilbene biosynthesis control is a prerequisite for further development of biotechnological approaches of commercial stilbene production as well as new plant protection strategies.

Current literature reveals that stilbene biosynthesis is regulated at the biochemical level by plant stress hormone signaling [14,15,16,17,18,19], ROS production [14,16,19], calcium signaling [14,15,20,21,22,23,24], and MAP kinase cascade [25]. After an external stimulus is perceived, activated receptors are proposed to convey the signal by activation of the MAPK cascade and calcium influx induction, leading to the activation of calcium sensor proteins [3]. Activated MAPK cascade and calcium sensor proteins could then induce ROS production and stimulate plant hormone signaling. These signaling events eventually lead to the activation of specific transcription factors (TFs) responsible for the induction of stilbene synthases (*STS*) genes and other genes responsible for stilbene biosynthesis [17,26,27]. However, at present, there is scarce information on the transcriptional regulation of stilbene biosynthesis.

It is known that biosynthesis of secondary metabolites in plants is regulated at the transcriptional level by multiple TFs, including MYC, MYB, WRKY and AP2/ERF TF families [28,29,30]. These TFs integrate internal and external cues and bind to certain *cis*-elements in gene promoter regions to induce or repress expression of the genes encoding enzymes responsible for biosynthesis of plant secondary metabolites [28,29,30]. The current literature presents multiple investigations that provide compelling evidence on the important roles of V-myb myeloblastosis viral oncogene homolog (MYB) TF family in the regulation of phenylpropanoid-derived secondary metabolites in plants (e.g., flavonoids, anthocyanines, and others), reviewed in [29]. Bearing in mind the contingency of biosynthesis of flavonoids and stilbenes, it is most probable that genes regulating the stilbene biosynthesis, such as *STS* genes, are also regulated by MYB transcription factors.

The first accession of the grapevine genome (×8.4) allowed the identification of 108 genes in the *Vitis vinifera* R2R3-MYB family [31,32]. According to the most recent data, the MYB R2R3 TF subfamily includes 134 annotated genes in *V. vinifera* [33]. The first findings on TF involvement in stilbene biosynthesis regulation revealed a positive role of *Myb14* and *Myb15* genes in the regulation of resveratrol biosynthesis in grapevine *V. vinifera* [26,27]. The results by Holl et al. (2013) [26] indicated that MYB14 and MYB15 TFs are involved in the induction of *STS29* and *STS41* transcription in *V. vinifera*. Using a one-hybrid yeast assay, it has been shown that MYB14 directly interacts with the *STS* promoter (Box-L5 motif) in vitro. A different study [27] confirmed that a transient overexpression of *MYB14* induced *STS* expression in grapevine leaves. Since the *STS* gene family comprises more than 30 functional *STS* genes [34], it is possible that stilbene biosynthesis in grapevine is regulated by more than two MYB TFs. Besides STSs, stilbene biosynthesis and modification pathway depend on other enzymes (glucosyltransferases, polyphenol oxidases, methyltransferases) whose expression could also involve regulation by MYB TFs. Recently, we have shown that, in addition to the *VaMyb14* and *VaMyb15* genes, the expression of *VaMYB9*, *40*, *60*, and *107* MYB TF genes was up-regulated in the tissues of wild grape *Vitis amurensis* Rupr. producing high stilbene amounts in response to UV or genetic transformation with a calcium sensor protein gene [35].

In this study, we show that the *VaMyb40* and *VaMyb60* genes function as potential positive regulators of stilbene biosynthesis in the grapevine by overexpressing the *Myb40* or *Myb60* genes in cell cultures of *V. amurensis*. The data provided evidence for the involvement of the *VaMyb40* and *VaMyb60* genes in positive regulation of stilbene biosynthetic processes in *V. amurensis*.

## 2. Results and Discussion

### 2.1. Genetic Transformation and Selection of the VaMyb-Transgenic Cell Lines

**Figure 1:**
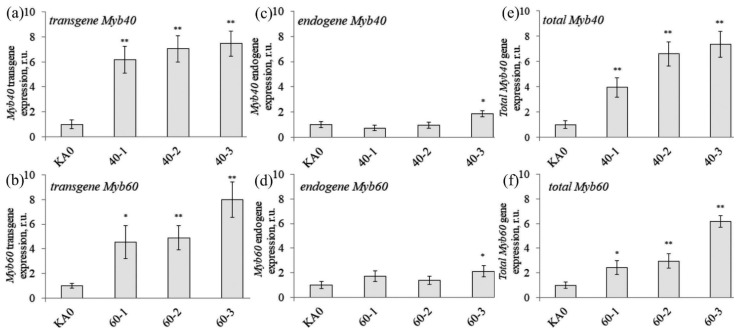
Quantification the transgene (**a**,**b**), endogenous (**c**,**d**), and total (**e**,**f**) mRNAs of the *VaMyb40* and *VaMyb60* genes in the transgenic callus cell lines of *Vitis amurensis* performed by quantitative RT-PCR. KA0—the control KA0 cell line of *V. amurensis* transformed with the vector harboring only the *npt*II selective marker; 40-1, 40-2, and 40-3—cell lines of *V. amurensis* transformed with the *VaMyb40* gene; 60-1, 60-2, and 60-3—cell lines of *V. amurensis* transformed with the *VaMyb60* gene. The data are presented as mean ± SE (two independent experiments with eight technical replicates). *, **—significantly different from the values of *Myb* expression in the control KA0 cell line at *p* ≤ 0.05 and 0.01 according to the Student’s t-test.

To establish cell cultures of *Vitis amurensis* Rupr. overexpressing the full-length *VaMyb40* and *VaCML60* genes, the V7 suspension culture of *V. amurensis* was incubated with *A. tumefaciens* strains bearing the pZP-RCS2-*VaMyb40/60*-*npt*II constructs for generation of the *VaMyb*-transgenic cells or the pZP-RCS2-*npt*II construct—for the control KA0 cell line, which contained only the kanamycin (Km) resistance gene *nptII*. All transgenes in the obtained constructs were under the control of the double cauliflower mosaic virus (CaMV 35 S) promoters. Then, we selected transgenic callus cell aggregates in the presence of 10–15 mg/L of Km for four months and established several Km-resistant independently obtained callus cell lines as described [23]. The selected transformed calli represented friable vigorously growing homogenous tissues, which did not undergo differentiation on the W_B/A_ medium supplemented with 6-benzylaminopurine (BAP) and α-naphthaleneacetic acid (NAA) in the dark. For further analysis, we used KA0 control transgenic cell line and six transgenic cell lines transformed with the *VaMyb40* and *VaMyb60* genes: three *VaMyb40*-transformed cell lines (40-1, 40-2, 40-3) and three *VaMyb60*-transformed cell lines (60-1, 60-2, 60-3) (Figure 1). After 2–3 months, the appearance of colored red areas was observed in the transgenic lines (Supplementary Figure S1). We found that these red tissue zone contained anthocyanins, which are described in detail below.

The *VaMyb*-transgenic callus cell lines were confirmed by qRT-PCR for expression of the *VaMyb40* and *VaMyb60* transgenes (Figure 1). All of the *VaMyb-*transformed cell lines actively expressed the transgenes (Figure 1a,b). The expression analysis of the endogenous *VaMyb40* and *VaMyb60* genes revealed that expression of the endogenous *VaMyb40* and *VaMyb60* was not affected in the 40-1, 40-2, 60-1, 60-2 cell lines in comparison with that in the control KA0 cell line (Figure 1c,d). However, the endogenous *VaMyb40* and *VaMyb60* expression was considerably increased in 1.9 and 2.1 times in the 40-3 and 60-3 lines. The mechanism of this increase is unclear, since a more common effect after transformation in such cases is a decrease in expression of endogenous gene counterpart observed due to gene silencing [36]. Perhaps, this increase was due to the fact that there was the highest value of total transgene and endogenous *VaMyb40* and *VaMyb60* in the 40-3 and 60-3 lines, and primers could have an additional glow, since one primer in a pair was the same. Then, we performed the analysis of the total transgene and endogenous *VaMyb40* and *VaMyb60* expression levels (Figure 1e,f). The total expression of *VaMyb40* and *VaMyb60* in all of the *VaMyb*-transformed calli considerably exceeded that in the KA0 control calli (Figure 1e,f). The total expression of the *Myb40* in the *VaMyb40*-transformed calli was elevated in 4.0–7.3 times (Figure 1e), and the total expression of the *Myb60* in the *VaMyb60*-transformed calli—in 2.5–6.2 times (Figure 1f), compared with KA0.

### 2.2. Stilbene Content and Biomass Accumulation in the Grapevine VaMyb-Transgenic Cell Lines

*V. amurensis* cell culture samples were collected from the 35-day-old calli for stilbene extraction and biomass analysis, because it has been shown that the highest content of stilbenes in the callus cell cultures was typical for the 35th day of cultivation [37]. In the Table 1, we presented fresh and dry biomass accumulation in the control KA0 and *VaMyb-*transformed callus cell lines of *V. amurensis*. Transformation of the *V. amurensis* calli with both the *VaMyb40* and *VaMyb60* genes degreased fresh weight (FW) accumulation in two cell lines out of three obtained, i.e., in 1.1–1.4 times and 1.1–1.2 times in the 40-1, 40-2, 60-2, and 60-3 lines, respectively, in comparison with FW of the KA0 cells (Table 1). However, the dry weight (DW) biomass levels of the *VaMyb*-transformed calli did not considerably differ from the DW levels of the KA0 cells (Table 1).

**Figure 2:**
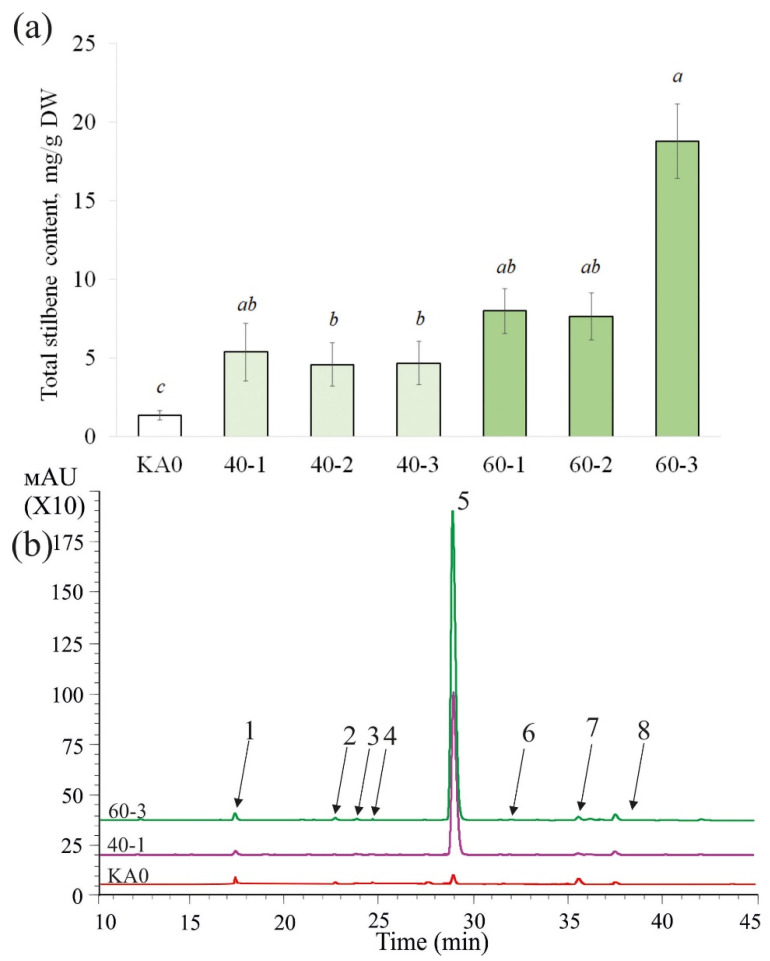
Total stilbene content ((**a**), mg per g of the dry weight (DW)) and a representative HPLC-UV profile ((**b**), 310 nm) of the callus cell lines of *Vitis amurensis* transformed with the *VaMyb40* or *VaMyb60* genes. KA0—control cell line transformed with the vector harboring only the *npt*II selective marker; 40-1, 40-2, 40-3—cell lines of *Vitis amurensis* transformed with the *VaMyb40* gene; 60-1, 2, 3—cell lines of *V. amurensis* transformed with the *VaMyb60* gene. *T*-resveratrol diglucoside (1), *t*-piceid (2), *t*-piceatannol (3), *cis*-piceid (4), *t*-resveratrol (5), *cis*-resveratrol (6), *t*-ε-viniferin (7), *t*-delta-viniferin (8). Means followed by the same letter were not different using one-way analysis of variance (ANOVA), followed by the Tukey HSD multiple comparison test (three independent experiments with four technical replicates). *p* < 0.05 was considered statistically significant.

Using HPLC, we determined the content and composition of stilbenes in the obtained *VaMyb*-transgenic cell lines. Overexpression of the *VaMyb60* gene led to a considerable increase in the content of stilbenes in all obtained transgenic lines in 5.9–13.9 times (Figure 2). The content of stilbenes was significantly increased in the cell lines of *V. amurensis* overexpressing the *VaMyb40* gene, although to a lesser extent than in the *VaMyb60-*overexpressing cell lines, i.e., in 3.4–4.0 times in comparison with stilbene content in KA0 (Figure 2).

The highest stilbene content and stilbene production were observed in the 60-3 *VaMyb60*-transgenic cell line and reached 18.8 mg/g DW and 148.7 mg/L, respectively (Figure 2; Table 1). To the best of our knowledge, this is one of the highest values of stilbene levels produced by plant cell cultures reported in the literature [3,38]. Stilbene content and production level in the 60-3 was in 13.2 times higher than stilbene production by the control cell culture KA0 (Table 1). It was also 4.2 times higher than stilbene production in transgenic grapevine cell lines transformed with the *VaCPK20* gene encoding a CDPK (up to 35 mg/L, [23]) and 1.1 times higher than stilbene production in grapevine cell lines transformed with the *VaCML65* gene encoding a calmodulin-like protein (up to 135.7 mg/L, [39]), but on 2.3% lower than that in the *rolB*-transgenic cell culture of *V. amurensis* (152 mg/L, [40]).

Overexpression of the *VaMyb40* and *VaMyb60* genes did not change the spectrum of detected individual stilbenes (Table 2). As in previously published works [39,41], we detected presence of eight stilbenes. Five stilbenes in all lines were well detectable (more than 0.06 mg/g DW): *t*-resveratrol diglucoside (1), *t*-piceid (2), *t*-resveratrol (3), ε-viniferin (4), δ-viniferin (5), and three stilbenes were usually detected in trace amounts (no more than 0.01 mg/g DW): *cis*-resveratrol (6), *cis*-piceid (7), *t*-piceatannol (8). The data revealed that the increase in the total content of stilbenes in the *VaMyb40*-transgenic cell lines was primarily due to a strong elevation in the content of *t*-piceid (in 2.5–5.4 times) and *t*-resveratrol (9.9–17.7 times) (Table 2). While the total content of stilbenes in the *VaMyb60*-transgenic cell lines was increased primarily due to a significant elevation in the content *t*-resveratrol (25.6–67.2 times), ε-viniferin (3.0–6.7 times), and δ-viniferin (2.4–4.6 times) (Table 2). *T*-resveratrol prevailed over other stilbenoid compounds (53.1–89.5% of all stilbenes) in both the *VaMyb40-* and *VaMyb60*-overexpressing cell cultures. The increase or decrease in the content of other stilbenes was not considerable (Table 2). Thus, our data indicate that overexpression of the *VaMyb40* and *VaMyb60* genes led to a marked increase in the content of stilbenes via a strong activation of *t-*resveratrol biosynthesis (Table 2).

**Figure 3:**
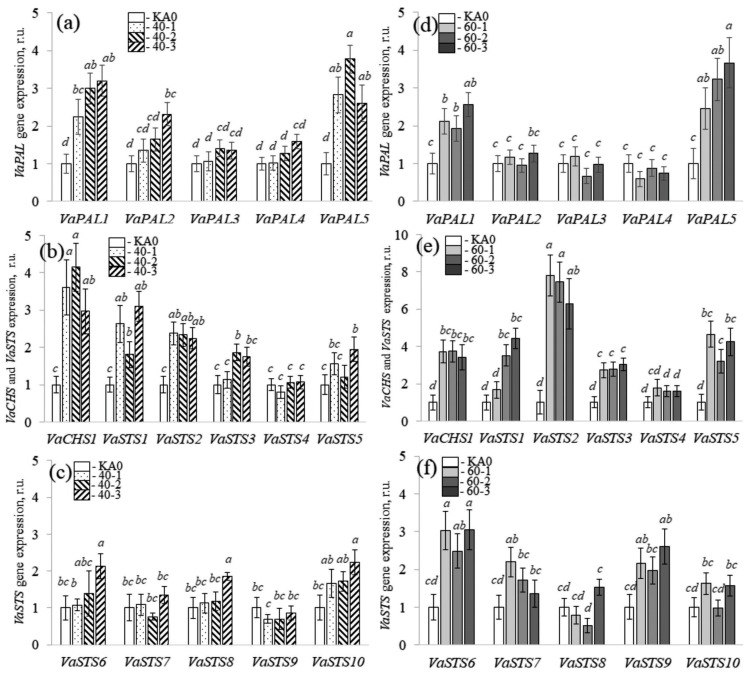
Quantification the *VaPAL1-5*, *VaCHS1*, and *VaSTS1-10* gene expression in the *VaMyb40-* (**a**–**c**) and *VaMyb60*-transgenic (**d**–**f**) cell lines of *Vitis amurensis* performed by quantitative RT-PCR. Means on each figure followed by the same letter were not different using one-way analysis of variance (ANOVA), followed by the Tukey HSD multiple comparison test (two independent experiments with eight technical replicates). *p* < 0.05 was considered to be statistically significant.

Then, it was important to verify whether the enhanced production of stilbenes in the grapevine cell cultures overexpressing the *VaMyb40* and *VaMyb60* genes was due to the activation of stilbene biosynthesis or to a reduction in the degradation of these compounds. For this purpose, we analyzed the expression of several important stilbene biosynthesis genes, including five phenylalanine ammonia-lyase (*PAL*) genes, one chalcone synthase (*CHS*), and ten stilbene synthase (*STS*) genes (Figure 3), which are known as important enzymes in stilbene biosynthesis [3].

The data obtained revealed that overexpression of the *VaMyb40* gene led to a considerable increase in the mRNA levels of the *VaPAL1* and *VaPAL5* (Figure 3a), *VaCHS1* (Figure 3b), and *VaSTS1* and *VaSTS2* genes (Figure 3b,c) in the *VaMyb40*-transgenic cell lines. Overexpression the *VaMyb60* gene led to a considerable increase in the mRNA levels of the *VaPAL1* and *VaPAL5* (Figure 3d), *VaCHS1* (Figure 3e), and *VaSTS1*, *2*, *3*, *5*, *6*, and *9* genes (Figure 3e,f) in the *VaMyb60*-transgenic cell lines. These results indicate that the enhanced content of stilbenes in the obtained *VaMyb40/60*-transgenic grape cells was associated with an activation of stilbene biosynthesis via a considerable increase in the expression of certain *PAL* and *STS* genes (Figure 3).

### 2.3. Anthocyanin Content in the Grapevine VaMyb-Transgenic Cell Lines

In red callus zones of the *Myb*-transformed *V. amurensis* cell lines, we detected the presence of five anthocyanins that were not present in the control KA0 cell line: cyanidin-3,5-o-diglucoside (1), delphinidin-3-o-glucoside (2), malvidin-3,5-o-diglucoside (3), cyanidin-3-o-glucoside (4), petunidin-3-galactoside (5) (supplementary Figure S2; Table S1). The total anthocyanin content in the *VaMyb40*-overexpressing lines was 0.035 ± 0.012 mg/g FW (40-1); 0.014 ± 0.003 mg/g FW (40-2); 0.013 ± 0.002 mg/g FW (40-3); and in the *Myb60*-overexpressing lines—0.009 ± 0.004 mg/g FW (60-1); 0.008 ± 0.004 mg/g FW (60-2); 0.008 ± 0.005 mg/g FW (60-3), respectively. Notably, we did not detect anthocyanins in in the control KA0 cell line. As can be seen from Table 1, the tissues of transgenic cultures dry out by about 22 times, thus, the content of anthocyanins in *VaMyb40*-overexpressing lines reached about 0.77 mg/g DW, and in *VaMyb60*-overexpressing lines—0.2 mg/g DW.

## 3. Materials and Methods

### 3.1. Plant Material and Cell Cultures

The V7 callus culture were initiated in 2017 from young stems of the wild-growing mature *V. amurensis* vines collected near Vladivostok as described [42]. The plant transgenic cell lines were established by *Agrobacterium*-mediated transformation as described [23].

### 3.2. Isolation and Sequencing of VaMyb40 and VaMyb60 Genes

To obtain the full-length cDNA of the *VaMyb40* and *VaMyb60* genes, we used primers (Table S2) designed to the beginning and to the end (from start to end codons) of the protein coding sequences based on the known *VvMyb40* and *VrMyb60* genes sequences in *V. vinifera* and *V. riparia,* respectively (GenBank accession number XM_019224778, XM_034837254). We were able to use primers designed for other grapevines, because we showed that the sequences of the *Myb* genes were very similar (supplementary Table S3). The generated PCR products of *VaMyb40* and *VaMyb60* genes were subcloned into a pJET1.2 using CloneJET PCR Cloninig Kit (ThermoFisher Scientific, Waltham, MA, USA) and sequenced using an ABI 3130 Genetic Analyzer (Applied Biosystems, Foster City, CA, USA) according to the manuphacturer’s instructions.

### 3.3. Overexpression of VaMyb40 and VaMyb60 in V. Amurensis Cell Cultures

Using *VaMyb40* and *VaMyb60* genes in pJET1.2 we performed PCR with the forward primer that contained a *Bgl*II and the reverse primer that contained a *Sal* I restriction site, which are underlined (Supplementary Table S2). The full-length cDNA of *Mybs* was cloned into the pSAT1 vector under the control of the double CaMV 35 S promoter [43] by the *Bgl*II and *Sal* I sites. Then, the expression cassette from pSAT1 with the *Myb* genes was cloned into the pZP-RCS2-*nptI*I vector [43] using the *Pal*AI (*Asc*I) sites. The pZP-RCS2-*npt*II construction also included the *npt*II gene under the control of the double CaMV 35 S promoter. The restriction enzymes were obtained from SibEnzyme (Novosibirsk, Russia). The independently transformed *VaMyb*-transgenic callus cell lines of *V. amurensis*, designated as 40-1, 40-2, 40-3 (*VaMyb40* gene) and 60-1, 60-2, 60-3 (*VaMyb60* gene), were obtained in 2021 by transformation of the V7 cell suspension with *A. tumefaciens* strain GV3101::pMP90 containing pZP-RCS2-*VaMybs*-*npt*II as described [23,38,44].

Transcript level of the *nptII* gene was analyzed using semiquantitative RT-PCR with the primes and PCR conditions described [45]. The absence of *A. tumefaciens* was confirmed by RT-PCR of the *VirB2* gene using primers listed in the Table S2 [45]. All transgenic cell lines were cultivated in 100 mL flasks with 20 mL of the solid Murashige and Skoog modified W_B/A_ medium [46] supplemented with 0.5 mg/L BAP, 2 mg/L NAA, and 8 g/L agar in the dark. For stilbene and anthocyanin analysis, the *V. amurensis* calli were cultivated at 35-day subculture intervals in the dark at 24–25 °C in test tubes (height 150 mm, internal diameter 14 mm) with 7–8 mL of the W_B/A_ medium.

### 3.4. Anthocyanin and Stilbene Analysis by High Performance Liquid Chromatography (HPLC) and Mass Spectrometry

Anthocyanin and stilbene content analysis was performed by the method HPLC-MS as described [45,47,48]. Briefly, for anthocyanins 100 mg fresh cells tissue were subsequently homogenized using a mortar and a pestle in 1 mL of 1% (*v/v*) hydrochloric acid in methanol. Then, shredded tissue was extracted for 1 d at 4 °C. For stilbenes 100 mg of the dried shredded cells tissue were extracted for 2 h at 60 °C in 3 mL of methanol. Then, anthocyanin and stilbene extracts were filtered through a 0.25-um nylon membrane for further analysis. Next, samples were separated on Shim-pack GIST C18 column (150 mm, 2.1-nm i.d., 3-_m part size; Shimadzu, Japan) the on HPLC LC-20AD XR analytical system (Shimadzu, Japan), equipped with an SPD-M20A photodiode array detector. Liquid chromatography-high-resolution mass spectrometry for qualification of all components was performed using a 1260 Infinity analytical system (Agilent Technologies, Santa Clara, CA, USA) as described [24,44].

The commercial standard cyanidin chloride, petunidin chloride, delphinidin chloride, malvidin chloride, *t*-resveratrol, *t*-piceid, *t*-piceatannol, ε-viniferin were obtained from Sigma-Aldrich (St. Louis, MO, USA) and used as the control.

### 3.5. RNA Isolation, Reverse Transcription and Quantitative Real-Time PCR (qRT-PCR)

Total RNA extraction was performed using the cetyltrimethylammonium bromide-based extraction as described [49]. Complementary DNAs were synthesized using the MMLV Reverse transcription PCR Kit with oligo(dT)15 (RT-PCR, Evrogen, Moscow, Russia) as described [50]. qRT-PCRs were performed using the real-time PCR kit (Evrogen) and SybrGreen I Real-time PCR dye (Evrogen) using total cDNAs as described [49,50].

The expression was calculated by the 2^−ΔΔCT^ method with two internal controls *VaGAPDH* and *VaActin1*, as described [51]. The qRT-PCR primers are listed in the Table S2. We used different primer sets for analyzing expression of the exogenous (transgene) and endogenous *VaMyb* genes (Supplementary Figure S3; Table S2).

### 3.6. Statistical Analysis

For quantification the *VaMyb*, *VaPAL*, and *VaSTS* genes expression, we used two independent experiments with ten technical replicates (five qPCR reactions normalized to *VaGAPDH* and five qPCR reactions—to *VaActin* gene in each independent experiment). We used three independent experiments with ten technical replicates in each experiment for callus tissue weight calculations and three independent experiments with two technical replicates for total stilbene measurement.

## 4. Conclusions

Previously, we found that two newly identified *VaMyb40* and *VaMyb60* genes were highly up-regulated in the wild-growing grapevine *V. amurensis* in response to UV irradiation and were suggested as promising candidates for playing important roles in stilbene biosynthesis [37]. Moreover, expression of the *VaMyb40* and *VaMyb60* genes was significantly increased in grapevine cell lines with elevated stilbene content as a result of overexpressing the *VaCPK20* and the calmodulin-like *VaCML65* genes [39]. In this paper, we investigated the effect of overexpressing *VaMyb40* and *VaMyb60* genes in callus cell cultures of *V. amurensis* on stilbene levels and composition. Both *VaMyb60* and *VaMyb40* gene overexpression activated stilbene biosynthesis and promoted stilbene accumulation, though effect of *VaMyb40* was lower. Overexpression of both *VaMyb40* and *VaMyb60* genes induced biosynthesis of *t*-resveratrol to a greater extent than biosynthesis of other individual stilbenes. Furthermore, we found that expression of stilbene biosynthesis genes, including *PAL* and *STS*, was considerably increased in the *VaMyb*-transgenic lines with elevated stilbene levels. Taken together, this indicates that these transcription factors primarily activate expression of stilbene biosynthesis-related genes and not the genes responsible for further stilbene metabolism.

The proposed model of the signaling pathway leading to stilbene biosynthesis induction with the involvement of *VaMyb40* or *VaMyb60* in this process was presented on the Figure S4. Briefly, after signal perception, calcium influx is induced, which then leads to the activation of calcium sensor proteins. Then, the signal is transferred via activation of mitogen-activated protein kinases (MAPK) cascade, CDPK, CML, hormone signaling, and some TFs, e.g., VaMyb40 or VaMyb06, which in turn lead to the transcriptional activation of the stilbene biosynthesis-related genes, such as *PAL* or *STS*.

In conclusion, the results are important for understanding the signaling pathways and mechanisms regulating biosynthesis of stilbenes and other phenolic metabolites and might be in demand for plant biotechnology and agriculture.

## Figures and Tables

**Table 1:** Biomass accumulation and total stilbene production in the cell lines of *Vitis amurensis* overexpressing the *VaMyb40* or *VaMyb60* genes.

Cell Line	Overexpressed *Myb* Gene	Fresh Weight, g/L	Dry Weight, g/L	Total Stilbene Production, mg/L
KA0	-	212.5 ± 9.7 ^a^	8.35 ± 1.03 ^a^	11.3 ± 1.8 ^d^
40-1	*VaMyb40*	177.1 ± 10.8 ^b^	7.94 ± 0.52 ^a^	42.7 ± 3.3 ^c^
40-2		157.4 ± 12.6 ^b^	7.42 ± 1.88 ^a^	33.9 ± 9.5 ^c^
40-3		210.9 ± 14.7 ^a^	8.92 ± 0.56 ^a^	41.7 ± 3.5 ^c^
60-1	*VaMyb60*	211.4 ± 10.3 ^a^	8.71 ± 0.93 ^a^	69.5 ± 7.4 ^b^
60-2		179.9 ± 9.6 ^b^	9.07 ± 0.42 ^a^	69.3 ± 4.3 ^b^
60-3		178.5 ± 14.7 ^b^	7.91 ± 1.08 ^a^	148.7 ± 19.2 ^a^

The callus tissue samples were harvested from the 35-day-old cultures (three independent experiments with eight technical replicates for weight calculations and three independent experiments with two technical replicates for total stilbene determination). Means followed by the same letter in one column were not different using one-way analysis of variance (ANOVA), followed by the Tukey HSD multiple comparison test.

**Table 2:** The content of individual stilbenes (mg per g of the dry weight (DW)) in the transgenic cell lines of *Vitis amurensis* transformed with *VaMyb40* or *VaMyb60* genes.

Cell Lines	Overexpressed *Myb* Gene	*t*-Resveratrol Diglucoside	*t*-Piceid	*t-*Piceatannol	*cis*-Piceid	*t*-Resveratrol	*cis*-Resveratrol	ε-Viniferin	δ-Viniferin
KA0	-	0.63 ± 0.09 ^a^	0.23 ± 0.08 ^bc^	0.01 ± 0.01 ^a^	0.01 ± 0.01 ^a^	0.25 ± 0.05 ^d^	0.01 ± 0.01 ^b^	0.06 ± 0.02 ^c^	0.14 ± 0.01 ^c^
40-1	*VaMyb40*	0.55 ± 0.08 ^a^	0.57 ± 0.27 ^b^	0.01 ± 0.01 ^a^	0.01 ± 0.01 ^a^	3.93 ± 1.36 ^bc^	0.01 ± 0.01 ^b^	0.09 ± 0.04 ^c^	0.22 ± 0.07 ^bc^
40-2		0.72 ± 0.08 ^a^	1.10 ± 0.29 ^ab^	0.01 ± 0.01 ^a^	0.01 ± 0.01 ^a^	2.53 ± 1.02 ^c^	0.01 ± 0.01 ^b^	0.06 ± 0.02 ^c^	0.16 ± 0.06 ^c^
40-3		0.66 ± 0.08 ^a^	1.25 ± 0.33 ^a^	0.01 ± 0.01 ^a^	0.02 ± 0.01 ^a^	2.48 ± 1.21 ^c^	0.01 ± 0.01 ^b^	0.07 ± 0.03 ^c^	0.19 ± 0.06 ^c^
60-1	*VaMyb60*	0.56 ± 0.11 ^a^	0.26 ± 0.08 ^bc^	0.01 ± 0.01 ^a^	0.01 ± 0.01 ^a^	6.41 ± 1.53 ^b^	0.16 ± 0.08 ^a^	0.18 ± 0.03 ^b^	0.39 ± 0.08 ^b^
60-2		0.61 ± 0.05 ^a^	0.12 ± 0.02 ^c^	0.01 ± 0.01 ^a^	0.01 ± 0.01 ^a^	6.39 ± 1.61 ^b^	0.01 ± 0.01 ^b^	0.19 ± 0.03 ^b^	0.34 ± 0.06 ^b^
60-3		0.73 ± 0.06 ^a^	0.19 ± 0.03 ^bc^	0.01 ± 0.01 ^a^	0.01 ± 0.01 ^a^	16.81 ± 2.39 ^a^	0.01 ± 0.01 ^b^	0.40 ± 0.05 ^a^	0.64 ± 0.07 ^a^

KA0—control cell line of *V. amurensis* transformed with the vector harboring only the *npt*II selective marker; 40-1, 2, 3—*V. amurensis* cell lines transformed with the *VaMyb40* gene; 60-1, 2, 3—*V. amurensis* cell lines transformed with the *VaMyb60* gene. The callus tissue samples were harvested from the 35-day old cell cultures. Means followed by the same letter in one column were not different using one-way analysis of variance (ANOVA), followed by the Tukey HSD multiple comparison test (three independent experiments with two technical replicates). *p* < 0.05 was considered statistically significant.

## Data Availability

The data presented in this study are available within the article and Supplementary Materials.

## Supplementary Materials

The following supporting information can be downloaded at: https://www.mdpi.com/article/10.3390/plants11151916/s1, Table S1: List of the stilbenes and anthocyanins derivatives identified in the methanol extracts of *Vitis amurensis* cells. Table S2: Primers used for amplification of *Vitis amurensis* cDNAs in PCR. Table S3: Comparison of the nucleotide sequences (BLAST) *Vitis amurensis VaMyb40* and *VaMyb60* genes with known *VvMyb40* and *VrMyb60* genes from *Vitis vinifera* and *Vitis riparia* respectively. The Genbank accession numbers are given in parentheses. Figure S1: *Vitis amurensis* cell lines used in the experiments. Figure S2: HPLC chromatograms of anthocyanins in *Vitis amurensis* in control KA0 cell culture (A) and cell line 40-1 overexpressed *VaMyb40* gene (detected at 530 nm). Figure S3: VaMyb40, VaMyb60, VvMyb40, and VvMyb60 genes sequences and primer design for PCR analysis. The colors of the primers match the primers shown in the supplementary table S1. Figure S4: Proposed model of the signaling pathway leading to stilbene biosynthesis induction and VaMyb40 and VaMyb60 functions in this process in the grapevine cells.
