# Supplementary material for: Effect of VaMyb40 and VaMyb60 Overexpression on Stilbene Biosynthesis in Cell Cultures of Grapevine Vitis amurensis Rupr
Source: Plants (Basel). 2022 Jul 24;11(15):1916. doi: 10.3390/plants11151916 (PMC9330820; doi:10.3390/plants11151916)

Supplementary Table S1. List of the stilbenes and anthocyanins derivatives identified in the methanol extracts of *Vitis amurensis* cells.

	Rt (min)	Elemental composition	Moll mass (g/M)	MS fragmentation		MS2 fragmentation		UV, λ max (nm)	Assignment
				Negative ion mod	Positive ion mode	Negative ion mod	Positive ion mode		
				[M-H]- (m/z)	[M+H]+ (m/z)	[M-H]-) (m/z)	[M+H]+) (m/z)		
Anthocyanins									
1[a]	13.1	C27H31O16	611.52	610	611	285	287;449	516	Cyanidin-3,5-O-diglucoside
2	14.3	C21H21O12	465.4	463	465	301	303	524	Delphinidin-3-O-glucoside
3	16.0	C32H27O13	655.6	654	655	491;329	331;493	528	Malvidin-3,5-O-diglucoside
4	17.4	C21H21O11+	449	447	449	284;211	287	516	Cyanidin-3-O-glucoside
5	19.2	C22H23O12+	479	478	479	314,299	317	525	Petunidin-3-galactoside
Stilbenes									
1[a]	17.1	C26H32O13	552.5	551	553	389;227	229;241	308	3,4'-diglucoside resveratrol
2	22.8	C20H22O8	390.388	389	391	227	229	310	*trans*-piceid
3	23.9	C20H22O8	390.388	389	391	227	229	290	cis-piceis
4	24.7	C14H12O4	244.246	243	245	225;201	147;133	320	trans-piceatannol
5	28.9	C14H12O3	228.25	227	229	185	142; 184; 114	310	*trans*-resveratrol
6	32.0	C14H12O3	228.25	227	229	185	142; 184; 114	290	*cis*-resveratrol
7	35.5	C28H22O6	454.47	453	455	359,347	349;343	320	*trans*-ε-Viniferin
8	37.7	C28H22O6	454.47	453	455	369;411;435	361;437	320	*trans*-D-Viniferin

[a]The peaks are numbered as shown in supplementary Figure S2 (anthocyanins) and Figure 2 (stilbenes).

Supplementary Table S2. Primers used for amplification of *Vitis amurensis* cDNAs in PCR.

cDNA	Primers names	Primers sequences, 5'-3'
**Primers for obtaining of the full *VaMybs* cDNA and generation the construction for plant cell transformation**		
Full *VaMyb40* cDNA obtaining	VaMyb40-nachS<br>VaMyb40-KonA	5'ATGGGAAGACAGCCTTGTTG,<br>5'TCAATGGCTATTTTCCAAGA,
Full *VaMyb60* cDNA obtaining	VaMyb60-nachS<br>VaMyb60-KonA	ATGGGAAGGCCTCCTTGCTG,<br>TCAGAATATTGGAGAGAGTT,
Overexpression of *VaMyb40*	VaMyb40-Bgl-pSATs<br>VaMyb40-Sal-pSATa	GCTC**AGATCT**ATGGGAAGACAGCCTTGTTG,<br>TCGA**GTCGAC**TCAATGGCTATTTTCCAAGA,
Overexpression of *VaMyb60*	VaMyb60-Bgl-pSATs<br>VaMyb60-Sal-pSATa	GCTC**AGATCT**ATGGGAAGGCCTCCTTGCTG,<br>TCGA**GTCGAC**TCAGAATATTGGAGAGAGTT,
**Primers for real-time PCRs**		
VaMyb40 transgene	VaMyb40-real-S<br>pSAT-term-A	ATCCTCATCTCTCCAAGATGAC,<br>GAGAGACTGGTGATTTTTGCG,
VaMyb40 endogene	VaMyb40-real-S<br>VaMyb40endo-real-A	ATCCTCATCTCTCCAAGATGAC,<br>CACAGAAGGGGAGACAGTATG,
VaMyb40 total	VaMyb40-real-S<br>VaMyb40-real-A	ATCCTCATCTCTCCAAGATGAC,<br>TCAATGGCTATTTTCCAAGAAGAAAAG,
VaMyb60 transgene	VaMyb60-real-S<br>pSAT-term-A	CTCTTGGATGAATCAGCAGC,<br>GAGAGACTGGTGATTTTTGCG,
VaMyb60 endogene	VaMyb60-real-S<br>VaMyb60endo-real-A	CTCTTGGATGAATCAGCAGC,<br>CTTCTTTTTATCATTTTATATCTCAAGCA,
VaMyb60 total	VaMyb60-real-S<br>VaMyb60-real-A	CTCTTGGATGAATCAGCAGC,<br>TCAGAATATTGGAGAGAGTTGATC,
VaPAL1 (EU659859)	VaPAL1-real-s<br>VaPAL1-real-a	5'CAGGACTTCACCTCAATGG,<br>5'GGGTTATCATTAACGGAGTTGA,
VaPAL2 (EU659860)	VaPAL2-real-s<br>VaPAL2-real-a	5'AAGGACTTCCCCTCAATGG,<br>5'GGGTTATCGTTCACTGAGTTTA,
VaPAL3 (EU659861)	VaPAL3-real-s<br>VaPAL3-real-a	5'TCCGAACATCTCCCCAGT,<br>5'ATCAAGGGATTGTCGTTTACC,
VaPAL4 (GQ443744)	VaPAL4-real-s<br>VaPAL4-real-a	5'AGCAAGATCGGTATGCTCT,<br>5'TCTATGGACTTCGTTGATGT,
VaPAL5 (GQ443745)	VaPAL5-real-s<br>VaPAL5-real-a	5'GTTGATGAAGCCTAAACAAG,<br>5'CCATGCGAATGACCTCAAT,
VaCHS1 (KT589834)	VaCHS-real-s<br>VaCHS-real-a	5'AAAGGGCACCACAGGGGAAG,<br>5'TCAGTGAGTCGATTGTGTAGCAAG,
VaSTS1 (EU659862)	VaSTS1-real-s<br>VaSTS1-real-a	5'CCAACCAAAGTCCAAGATCA,<br>5'CCTTCTAACCGATGTTTCAAGA,
VaSTS2 (EU659863)	VaSTS2-real-s<br>VaSTS2-real-a	5'CGGTGCGGATTACAAACTC,<br>5'CACCCTTGATGGTACAACAT,
VaSTS3 (EU659864)	VaSTS3-real-s<br>VaSTS3-real-a	5'GTCAGCCTAAATCGAAGATCAC,<br>5'TCTTCTAACAGATGGTTCGAGG,
VaSTS4 (EU659865)	VaSTS4-real-s<br>VaSTS4-real-a	5'ACAACCTCTGGTGTAGAAAT,<br>5'CTGACCGATGTTTCAAGGC,
VaSTS5 (EU659866)	VaSTS5-real-s<br>VaSTS5-real-a	5'CCTCGAACCATCTGTTAGAAGA,<br>5'TCTCCGCAAGATCCTTAGC,
VaSTS6 (EU659867)	VaSTS6-real-s<br>VaSTS6-real-a	5'CCTCAGGTGTAGAAATGCC,<br>5'CATCACTCTTCTAACAGATGG,
VaSTS7 (EU659868)	VaSTS7-real-s<br>VaSTS7-real-a	5'GCAGCACTGAAGGCACTTAA,<br>5'ATTTCTACACCTGACGTTGT,
VaSTS8 (GQ443746)	VaSTS8-real-s<br>VaSTS8-real-a	5'GCCTCGAACCATCTGTCA,<br>5'AAGATCCTTAGCTGTTCGA,
VaSTS9 (GU266256)	VaSTS9-real-s<br>VaSTS9-real-a	5'GGCAGCCTAAGTCCAAGATTA,<br>5'AGTCTGCACCGGGCATTTC,
VaSTS10 (JQ780328)	VaSTS10-real-s<br>VaSTS10-real-a	5'GCAGGAGCACGGGTTCT,<br>5'AGTCCAAAGCAATTTCAGAA,
VaActin1	*VaActin*-realS	5'GTATTGTGCTGGATTCTGGTGA,

(DQ517935)	*VaActin*-realA	5'GCAAGGTCAAGACGAAGGATAG,
VaGAPDH (AM437491)	*VaGAPDH*-realS<br>*VaGAPDH*-realA	5'CACTGAAGATGATGTTGTTTCC,<br>5'GCTATTCCAGCCTTGGCAT,
**Primers for semiquantitative RT-PCR**		
*npt*II (AY818371)	NPTII-SER-S<br>NPTII-KON-A	5'ATTCGACCACCAAGCGAAAC,<br>5'TCAGAAGAACTCGTCAAGAA,
VirB2 (KY000054)	VIRB2-S<br>VIRB2-A	5'ATGCGATGCTTTGAAAGATACCG,<br>5'TTAGCCACCTCCAGTCAGCG

Supplementary Table S3. Comparison of the nucleotide sequences (BLAST) *Vitis amurensis VaMyb40* and *VaMyb60* genes with known *VvMyb40* and *VrMyb60* genes from *Vitis vinifera* and *Vitis riparia* respectively. The Genbank accession numbers are given in parentheses.

	VaMyb40	*VaMyb60*	*VvMyb40*	*VrMyb60*
VaMyb40	**-**	52%	**100%**	52%
VaMyb60	52%	**-**	52%	**99.2%**
VvMyb40 (XM_019224778)	**100%**	52%	**-**	52%
VrMyb60 (XM_034837254)	52%	**99.2%**	52%	**-**

Supplementary Figure S1. *Vitis amurensis* cell lines used in the experiments.

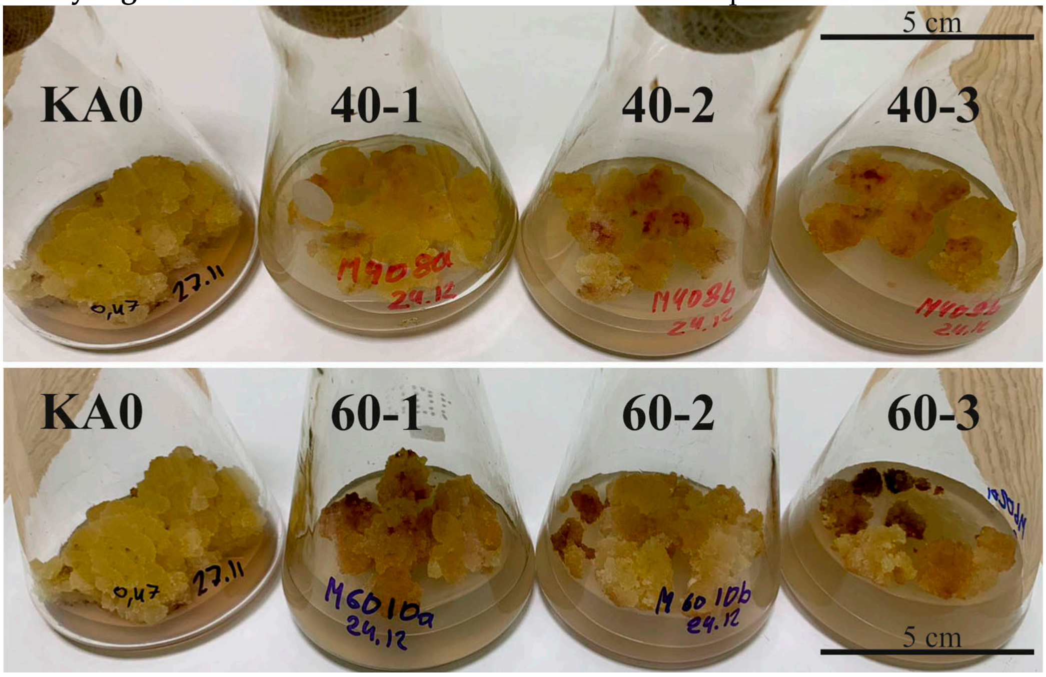

Supplementary Figure S2. HPLC chromatograms of anthocyanins in *Vitis amurensis* in control KA0 cell culture (A) and cell line 40-1 overexpressed *VaMyb40* gene (detected at 530 nm). Cyanidin-3,5-O-diglucoside (1), delphinidin-3-O-glucoside (2), malvidin-3,5-O-diglucoside (3), cyanidin-3-O-glucoside (4), petunidin-3-galactoside (5).

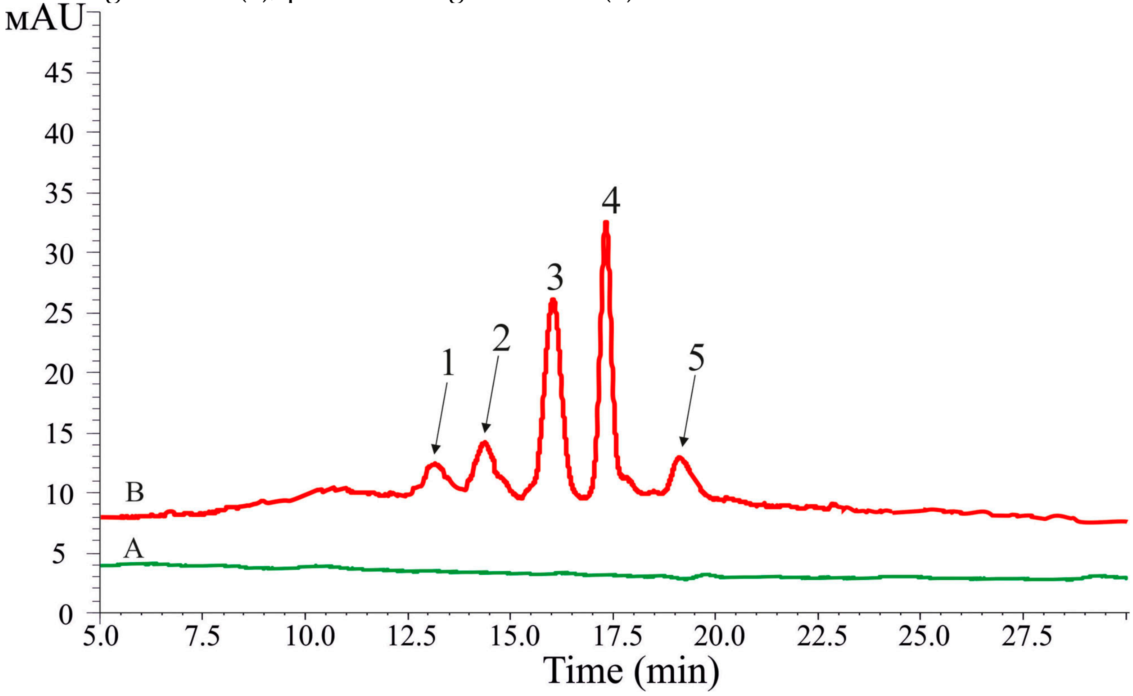

Supplementary Figure S3. *VaMyb40*, *VaMyb60*, *VvMyb40*, and *VvMyb60* genes sequences and primer design for PCR analysis. The colors of the primers match the primers shown in the supplementary table S1.

>VaMyb40 (s1719-1)

ATGGGAAGACAGCCTTGTTGTGACAAGGTTGGGTTGAAGAGAGGTCCATGGACAATTGAGGAAGATCACAAACTCAT
GAGCTTCATCCTCAATAATGGTATCCAATGCTGGAGAATGGTTCCCAAGCTTGCAGGATTGTTGAGATGTGGAAAGAG
TTGCAGACTAAGATGGATCAATTATCTGAGGCCGGATCTTAAGAGGGGCGCACTATCAGAATTGGAAGAGAATCAGA
TAATCCAACTTCATTCACGTCTTGGTAACAGGTGGTCTAAGATTGCCTCACACTTCCCTGGTCGCACGGATAATGAAAT
CAAGAACCACTGGAATACACGAATCAAGAAGCGGCTAAAGCAACTGGGGTTAGACCCAGTGACCCACAAACCAATCG

AGGAAAAGGATGAGGTCAATGAGAAAAATGACACGGTTCCAGACTCTGCTTCGTCAAAACAGCAAGAGGAAAGTAAT
GGTGAGAGGGGTGATACTCTGGACACAGTACAAGGGAAGCAAACTGGAGGGGAAATGACCAGCCATGAAGCAAGTA
CTGACGATCTTTTAAATGAAATGTTGGGTGGAAGCTCGGACTTGGAGTTGGGACTACTGATGAACCAGCAAACCAAAA
CCTCATCAAACACCTACAGTCCCTCATTTTCTCTAGAAGATTCTCTCAACCCTTCCATGGCTGAATCCTCATCTCTCCAA
GATGACTGCTTACAGCAATGGGTTGATTGTACAGTGGACTCTCTACTCTCATGGGACAACTTCAATCATCTAGAACAA
GAGCTTTTCTTCTTGGAAAATAGCCATTGAAT

>VvMyb40 (DEFINITION PREDICTED: *Vitis vinifera* myb-related protein 315-like (LOC109123830), mRNA), ACCESSION, XM_019224778

TTCCATGTAAAATTTCTACAAACGAAGGCACTGTCTATTGTCTCAATAAGGTGGTGGATATTTTATTATTACATATCCT
TGATGCCTTTGGGTTATCAACTCTAAATGCTCCCCCCATTCAATCATGTATCTCCCCCCTTAAACTACATTTTACTTTTA
AAAATGTTGGGATAACCAGCTGGCAGCATCTTTGTTTGGGTTATAAGGCCCCTTTTCGGGCTGTCTTGCTTATTGTTGT
TGTCACAAATCCCACCATTTCTCTCCTTTCTTTTCCCTGTGCCAAGTCTGGAAGATGGGAAGACAGCCTTGTTGTGACA
AGGTTGGGTTGAAGAGAGGTCCATGGACAATTGAGGAAGATCACAAACTCATGAGCTTCATCCTCAATAATGGTATCC
AATGCTGGAGAATGGTTCCCAAGCTTGCAGGATTGTTGAGATGTGGAAAGAGTTGCAGACTAAGATGGATCAATTATC
TGAGGCCGGATCTTAAGAGAGGCGCACTATCAGAAGTGGAAGAGAATCAGATAATCCAACTTCATTCACGTCTTGGTA
ACAGGTGGTCTAAGATTGCCTCACACTTCCCTGGTCGCACGGATAATGAAATCAAGAACCACTGGAATACACGAATCA
AGAAGCGGCTAAAGCAACTGGGGTTAGACCCAGTGACCCCACAAACCAATCGAGGAAAAGGATGAGGTCAATGAGAA
AAATGACACGGTTCCAGACTCTGCTTCGTCAAAACAGCAAGAGGAAAGAAATGGTGAGAGGGGTGATACTCTAGACA
CAGTACAAGAGAAACAAACTGGAGGGGAAATGACCAGCCATGAAGCAAGTACTGACGATCTTTTAAATGAAATGTTG
GATGGAAGCTCGGACTTGGAGTTGGGACTACTGATGAACCAGCAAACCAAAACCTCATCAAACACCTACAGTCCCTC
ATTTTCTCTAGAAGATTCTCTCAACCCTTCCATGGCTGAATCCTCATCTCTCCAAGATGACTGCTTACAGCAATGGGTT
GATTGTACAGTGGACTCTCTACTCTCATGGGACAACTTCAATCATCTAGAACAAGAGCTTTTCTTCTTGGAAAATAGCC
ATTGAACATACTGTCTCCCCTTCTGTGCCTTAGTGACTTTAGTTTCACCCATCAGGATGATCATCGCTCATGCAATAAA
AAATTTCTCAAAAAGAAAGTGTAAATCATATTATTTGATCTAGTGTTTTCCAATATGACATGGATTCCTCCTCCACCT
TATTGTTTTGCTGTCAATCATTGAAACTTGTGTTTTATTGGAGTCATGTTTTAATCAACCTGTATTTGTTTATAACAATC
TCTTGTTCTTAATTA

>VaMyb60 (s1719-6)

ATGGGAAGGCCTCCTTGCTGTGATAAAGTTGGTATCAAGAAGGGTCCTTGGACCCCAGAAGAGGACATCATCTTGGTC
TCCTATATCCAAGAGCATGGCCCCGGAAATTGGAGATCAGTGCCTACAAACACCGGGCTGCTGAGGTGTAGCAAGAG
TTGCAGGCTTAGATGGACTAATTACCTTAGACCGGGGATTAAGCGCGGTAACTTCACTCCCCATGAAGAAGGAATGAT
CATCCATCTACAAGCCCTATTGGGTAACAAATGGGCTGCCATAGCTTCATACCTCCCTCAAAGAACTGATAATGATAT
AAAGAATTATTGGAACACTCACTTGAAGAAGAAGATCAAGAAGTTTCAGTCAGCTTTGAGCCCCCATATGGCCTCAGA
TTCAACCACAAGCACCTGTACTAATCATCAATTTGTACCAAGGAGCTATGCTGGTGATGATCATCACAGAAGGGGTAG
TTCTTTTGAGGTCATCAATGGCCATTCCTCGGCTCACCCGAGCCTAAACAGCCCGATCTCGACGTATGCCTCCAGCACT
GAGAACATCTCAAGGCTACTAGAAGGTTGGATGAGGTCCTCCCCAAAGGCCACCAAGGAGAAACTGCACCAAAACAG
CAGCTTGGAAGAAGGTAGTATCGATATGACCGGAAACTCCATGGCGGTGGCGGCGGTGACTTCTGTCCAGTGTTACAG
GCCTAAGTTGGAGCAAGGAGGGGGCGAGTTGGTCGCCAACGACGAGTTTGAGTCCATGCTTGAGTACGAAAACCTGA
ATGATGATCATCATCAGACTACTGATACTACTATTCCAAGTGATGATCATGATCATGATCATGAGATGAAGATGGATC
ATGATCAGAAGAAGCACAACCCTCCTCTATCATTTCTTGAGAAATGGCTCTTGGATGAATCAGCAGCTCAAGGAGAGG
AGATGATGGATCAACTCTCTCCAATATTCTGA

>VvMyb60 (ACCESSION XM_034837254, VERSION XM_034837254.1)

AGAGGTTTTATGAGTCACTCTTAGTATATAAACACCCTCACATGGTGATATAATACTATTTGAAAGGCACGAAGAGAT
AGATAGAGAGAGAGAGAGAGAGAAAGACAGACAGACAGAGAGAGAGAGAGAGAGAGAGAGAGTTTGGGATGAAAACAAG
CCATGGGAAGGCCTCCTTGCTGTGATAAAGTTGGTATCAAGAAGGGTCCTTGGACCCCAGAAGAGGACATCATCTTGG
TCTCCTATATCCAAGAGCATGGCCCCGGAAATTGGAGATCAGTGCCTACAAACACCGGGCTGCTGAGGTGTAGCAAG
AGTTGCAGGCTTAGATGGACTAATTACCTTAGACCGGGGATTAAGCGCGGTAACTTCACTCCCCATGAAGAAGGAATG
ATCATCCATCTACAAGCCCTATTGGGTAACAAATGGGCTGCCATAGCTTCATACCTCCCTCAAAGAACTGATAATGAT
ATAAAGAATTATTGGAACACTCACTTGAAGAAGAAGATCAAGAAGTTTCAGTCAGCTTTGAGCCCCCATATGGCCTCA
GATTCAACCACAAGCACCTGTACTAATCATCAATTTGTACCAAGGAGCTATGCTGGTGATGATCATCACAGAAGGGGT
AGTTCTTTTGAGGTCATCAATGGCCATTCCTCGGCTCACCCGAGCCTAAACAGCCCGATCTCGACGTATGCCTCCAGCA
CTGAGAACATCTCAAGGCTACTAGAAGGTTGGATGAGGTCCTCCCCAAAGGCCACCAAGGAGAAACTGTACCAAAAC
AGCAGCTTGGAAGAAGGTAGTATCGATATGACCGGAAACTCCATGGCGGTGGCGGCGGTGACTTCTGTCCAGTGTTAC
AGGCCTAAGTTGGAGCAAGGAGGGGGCGAGTTGGTCGCCAACGACGAGTTTGAGTCCATTCTTGAGTACGAAAACCT
GAATGATGATCATCATCAGACTACTGATACTACTATTCCAAGTGATGGTCATGATCATGATCATGATCATGAGATGAA
GATGGATCATGATCAGAAGAAGCACAACCCTCCTCTATCATTTCTTGAGAAATGGCTCTTGGATGAATCAGCAGCTCA
AGGAGAGGAGATGATGGATCAACTCTCTCCAATATTCTGATAAATTAAAGAAAGAAAACCATTGTATATCTTTCTCTT
TTTCTTTATGCTTGAGATATAAAATGATAAAAAGAAGAAGAAGAAGAAAAAAGGAAAAAACAAAAAAAAAGAAAAA
AAAAAGAGAGTTTAATCTTCTTAGGTTTAAAGGTGATCTTGTTGGGGTTCTGAAAAGTTGGGATCTCTTTGCAAAATGT
AACCTTGAATGTAATTCTGAGTGAATTATCATCCTGAGTGAACTCAGTTTTCATGTGCTTTTAATTATTGCTCTAAAAT
AGGGGGTAACTTAGTGATAATTCTAATGAAAATTATATTCCAACAA

Supplementary Figure S4. Proposed model of the signaling pathway leading to stilbene biosynthesis induction and VaMyb40 and VaMyb60 functions in this process in the grapevine cells. Ultraviolet (UV), elicitors, pathogen effectors, and other environmental cues are perceived by specific receptors. Recognition of an external signal leads to sustained calcium influx, activation of calcium sensors, MAPK cascades, which promotes plant hormone signaling and transcription factors (TF) activation including VaMyb40 and VaMyb60. TF activate transcription of the genes responsible for stilbene biosynthesis. CDPK – calcium-dependent protein kinases; CML – calmodulin-like proteins; MAPK – mitogen activated protein kinases; TF - transcription factors.

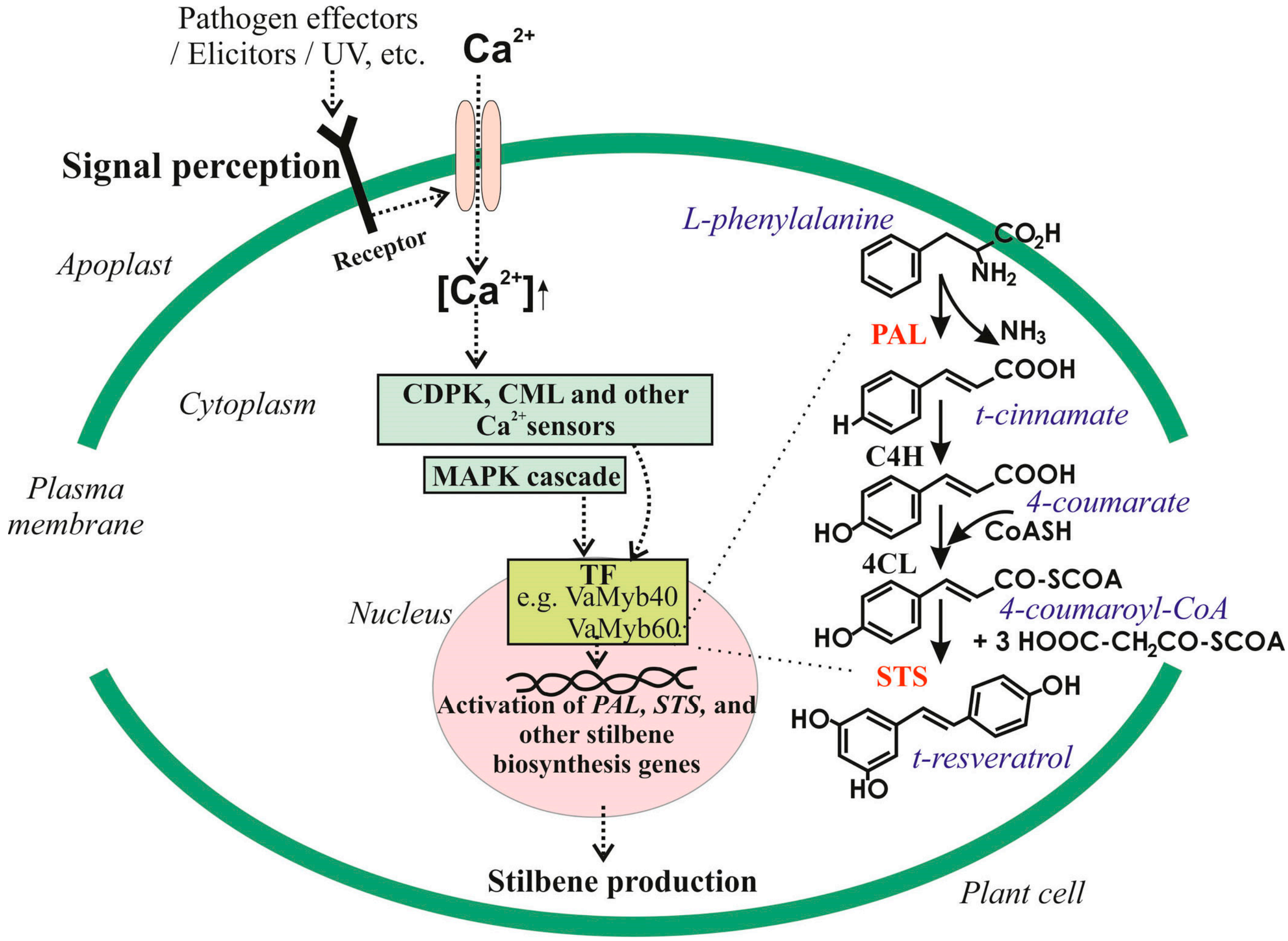

Supplement: Supplementary file 1 [file plants-11-01916-s001.zip › plants-1799881-supplementary.pdf]
